# Enhanced energy storage in supercapacitors using R-TiO_2_ nanotube and graphene-based electrodes

**DOI:** 10.1039/d5ra07750h

**Published:** 2026-02-06

**Authors:** Sensu Tunca, Iqra Rabani, Karolien De Wael

**Affiliations:** a Antwerp Engineering, Photoelectrochemistry & Sensing (A-PECS), University of Antwerp Groenenborgerlaan 171 2020 Antwerp Belgium karolien.dewael@uantwerpen.be; b NANOlight Center of Excellence, University of Antwerp Groenenborgerlaan 171 2020 Antwerp Belgium

## Abstract

Conventional thin-film supercapacitors are limited by low energy density and poor charge balance between electrodes, restricting their integration into miniaturized electronic devices. In this study, reduced TiO_2_ nanotubes (R-TiO_2_ NTs) were fabricated *via* a straightforward anodization process followed by electrochemical reduction (self-doping) and further decorated with Ni(OH)_2_ nanospheres. These R-TiO_2_ NTs/Ni(OH)_2_ NSs electrodes were employed as both positive and negative electrodes for symmetric supercapacitors, and as positive electrodes in asymmetric configurations. To develop a suitable negative electrode, few-layer graphene (FLG) and graphene nanoplatelets (GNP) were combined, and the optimal FLG/GNP weight ratio was identified to balance charge storage. This electrode design enabled the fabrication of an asymmetric supercapacitor (ASC) with significantly enhanced energy storage performance. The superior performance of the ASC is attributed to a synergistic charge storage mechanism, where surface-controlled pseudocapacitive reactions of Ni(OH)_2_ nanosheets complement the double-layer capacitance of the FLG-GNP electrode, ensuring rapid charge–discharge kinetics, high rate capability, and excellent cycling stability. The ASC achieved an areal capacitance of 118.26 mF cm^−2^ and an energy density of 42.05 µWh cm^−2^ at 0.25 mA cm^−2^, compared to 19.38 mF cm^−2^ and 6.89 µWh cm^−2^ for the symmetric device. This work demonstrates a promising strategy for high-performance, scalable micro-supercapacitors with potential applications in flexible and miniaturized electronics.

## Abstract 2

A high-performance, scalable micro-supercapacitor delivering enhanced capacitance, energy density, and long-term cycling stability.

## Graphical abstract

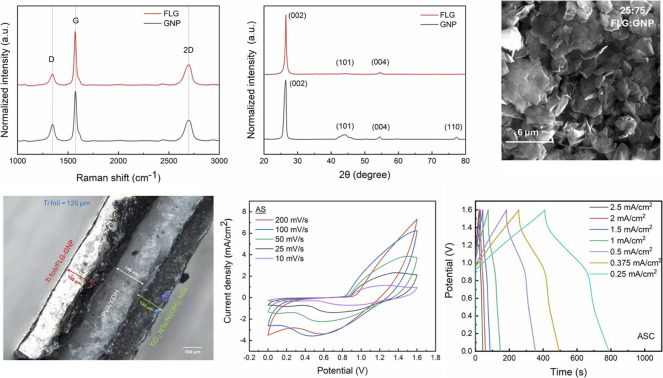

## 1 Introduction

With the rapid global increase in energy demand, supercapacitors (electrochemical capacitors) have gained significant importance.^1,2^ As society advances toward smart technologies and widespread electrification, supercapacitors are becoming essential for energy storage.^3,4^ Their high power density (*i.e.*, how fast the energy can be delivered) makes them increasingly critical for portable electronics, hybrid vehicles, and renewable energy systems.^4,5^ However, their widespread application is limited by low energy density (*i.e.*, how much energy is stored).^6^ Moreover, areal metrics serves as a key performance indicator, as the compact design of current electronics limits the amount of active material that can be integrated.^7^ Historically, supercapacitors have evolved to bridge the performance gap between conventional capacitors and batteries, driving significant research toward their miniaturization, enhanced performance, and integration into advanced technologies.^8^ Unlike conventional capacitors, which rely primarily on electrostatic charge storage, supercapacitors employ electrode materials with high surface areas, such as nanostructured materials, to substantially increase capacitance and overall performance.^9,10^

Supercapacitors can be configured in either symmetric or asymmetric designs, depending on the composition of the electrode materials. The mechanisms governing charge storage further differentiate their performance.^4^ In electrical double-layer capacitors (EDLCs), charge accumulation occurs *via* the physical adsorption of electrolyte ions onto the electrode surface, enabling rapid charge–discharge cycles and high power density but relatively low energy storage compared to batteries.^11^ In contrast, pseudocapacitors store charge through fast, reversible faradaic redox reactions, resulting in higher capacitance and improved energy and power densities.^12,13^ Despite the high power capability of supercapacitors, low energy density remains a key limitation, driving research toward strategies that enhance energy storage without compromising power performance,^10,14,15^

In this regard, titanium dioxide nanotubes (TiO_2_ NTs) have attracted considerable attention in supercapacitor research due to their highly ordered architecture, mechanical and chemical stability, large surface area, and unidirectional electron pathways.^15,16^ However, their high electrical resistivity limits charge storage performance.^16^ In our previous study, an electrochemical reduction approach was employed to fabricate reduced TiO_2_ NTs (R-TiO_2_ NTs), which exhibited significantly enhanced electrical conductivity and areal capacitance (1.18 mF cm^−2^) compared to pristine TiO_2_ NTs (0.03 mF cm^−2^). R-TiO_2_ NTs were subsequently used to develop pseudocapacitive electrodes by depositing Ni(OH)_2_ nanospheres (R-TiO_2_ NTs/Ni(OH)_2_ NSs), resulting in a remarkable 3700-fold increase in areal capacitance (305.91 mF cm^−2^) relative to TiO_2_ NTs/Ni(OH)_2_ NSs electrodes (0.081 mF cm^−2^).^17^

Building upon this foundation, the present work investigates R-TiO_2_ NTs/Ni(OH)_2_ electrodes in both thin-film symmetric supercapacitors (SSC) and asymmetric supercapacitors (ASC) to identify the most efficient device architecture and elucidate charge storage mechanisms for high energy density. For supercapacitor fabrication, activated carbon is one of the most widely used electrode materials for electrical double-layer capacitors, owing to its high specific surface area, low cost, and hierarchical pore structure. However, its intrinsic high resistance can limit device performance without conductive additives.^18,19^ To address this, few-layer graphene (FLG) and graphene nanoplatelets (GNP) were selected as conductive additives.^20,21^ FLG provides efficient electron transport channels and a high surface area, making it suitable for high-energy and high-power supercapacitors.^22,23^ GNPs, consisting of short stacks of graphene sheets, provide high surface area, excellent electrical conductivity, and mechanically robust structures. These characteristics facilitate rapid ionic transport and enhance charge storage stability in electrochemical devices.^24,25^ To optimize the performance of carbon-based electrodes, the influence of the ratio between FLG and GNP on electrode capacitance, charge retention, and stability was systematically investigated.

Using these insights, thin-film SSC and ASC devices were fabricated based on R-TiO_2_ NTs/Ni(OH)_2_ NSs electrodes, with FLG-GNP electrodes serving as the counter electrode for the ASC. Electrochemical characterization through cyclic voltammetry (CV), galvanostatic charge–discharge (GCD), and electrochemical impedance spectroscopy (EIS) identified the optimal FLG/GNP composition, revealing the contributions of each component to charge storage and capacitance retention. The optimized ASC (R-TiO_2_ NTs/Ni(OH)_2_ | Ti foil/FLG-GNP) demonstrated superior performance compared to the SSC (R-TiO_2_ NTs/Ni(OH)_2_ | R-TiO_2_ NTs/Ni(OH)_2_), achieving higher areal capacitance and energy density by combining pseudocapacitive and EDLC mechanisms. These results highlight the potential of R-TiO_2_ NTs-based thin-film supercapacitors and provide a valuable contribution to the limited studies on TiO_2_ NTs for advanced energy storage applications.

## 2 Experimental details

### 2.1 Fabrication of the R-TiO_2_ NTs/Ni(OH)_2_ NSs electrodes

Preparation of the R-TiO_2_ NTs/Ni(OH)_2_ NSs electrodes was performed according to our previous paper.^17^ Multi-range DC power supply (B&K Precision, model 9104) was used for the fabrication of the TiO_2_ NTs *via* the electrochemical anodization method. A typical two-step electrochemical anodization method was employed in a two-electrode configuration using Ti foil (1 cm × 1 cm × 0.125 mm) as the anode, and a platinum (Pt) sheet as the cathode. The back side of the Ti foil was covered with Kapton tape to confine NT growth to an area of 1 × 1 cm^2^. The electrolyte consists of 0.3 wt% NH_4_F and 1 vol% H_2_O in 25 mL ethylene glycol (EG). During the first anodization step, a bias of 40 V was applied for 1 h at room temperature. The Ti foil was then immediately rinsed with DI water and ultrasonicated in DI water for 20 min to remove the initially formed TiO_2_ NTs leaving behind a textured template layer. After drying in air, a second anodization was carried out by applying a 40 V bias for 2 h. Following the completion of the electrochemical anodization, the electrode was rinsed with DI water and dried in air at room temperature. Finally, as-prepared amorphous TiO_2_ NTs were annealed at 450 °C for 2 h (2 °C min^−1^) in air to obtain anatase TiO_2_ NT electrodes. Metrohm Autolab, PGSTAT204 was used during the electrochemical reduction of TiO_2_ NTs, and electrochemical deposition of Ni(OH)_2_ NSs onto the R-TiO_2_ NTs. Initially, TiO_2_ NTs were electrochemically reduced using a three-electrode configuration. Anatase TiO_2_ NTs were used as the working electrode, Pt sheet as the counter electrode and saturated calomel electrode (SCE) as the reference electrode. Electrochemical reduction was performed at a potential of −1.6 V for 10 min in a 0.1 M Na_2_SO_4_ aqueous electrolyte. Similarly, electrodeposition of Ni(OH)_2_ NSs was conducted in a three-electrode configuration where R-TiO_2_ NTs served as the working electrode, and the electrolyte was an aqueous solution of 0.1 M nickel acetate (Ni(CH_3_COO)_2_·4H_2_O). Prior to electrodeposition, R-TiO_2_ NT electrode was immersed in the electrolyte under constant stirring for 30 min to ensure complete wetting of the surface. The Ni(OH)_2_ NSs were then deposited by applying a constant potential of −0.9 V for 600 s. After deposition, the R-TiO_2_ NTs/Ni(OH)_2_ NSs electrodes were rinsed thoroughly with DI water and dried in air.

### 2.2 Fabrication of the Ti foil/FLG-GNP electrodes

All Ti foil/FLG-GNP electrodes were fabricated by drop casting FLG-GNP mixtures onto 1 × 1 cm^2^ Ti foil substrates. Prior to deposition, Ti foils were cleaned by ultrasonication for 15 min each in acetone, ethanol, and deionized water and subsequently dried in air. The electrode composition was fixed at 80 wt% active material (FLG and/or GNP), 10 wt% PVDF, and 10 wt% AC (80 : 10 : 10 by weight). Five different mixtures of FLG and GNP were prepared with varying ratios of FLG : GNP (100 : 0, 75 : 25, 50 : 50, 25 : 75, and 0 : 100, wt%). The active material fraction (FLG : GNP) corresponds to 80% of the total electrode material mass. All electrode material was dispersed in *N*-methyl-2-pyrrolidinone and ground using an agate mortar until a homogeneous slurry was obtained. The slurry was drop-cast onto Ti foils, followed by drying at 100 °C in air. The average thickness of the electrode coating was approximately 30 µm.

### 2.3 Fabrication of the symmetric and asymmetric thin film supercapacitors

SSC and ASC thin-film SCs were assembled using R-TiO_2_ NTs combined with Ni(OH)_2_ NSs and FLG-GNP as active materials. For the SSC, both the positive and negative electrodes consisted of R-TiO_2_ NTs/Ni(OH)_2_ NSs. For the ASC, the positive electrode was R-TiO_2_ NTs/Ni(OH)_2_ NSs, whereas the negative electrode was Ti foil coated with FLG-GNP. In both device configurations, Ti foil functioned as a current collector, and PVA/KOH gel was used as electrolyte and separator. The gel electrolyte was prepared by dissolving 1 g of PVA in deionized water and heating the mixture at 90 °C on a hot plate until a clear solution was obtained. Separately, 1 g of KOH was dissolved in 10 mL of water, and this solution was added dropwise to the PVA solution under constant stirring. The resulting PVA/KOH gel was cooled to room temperature and cast into Petri dishes to form films with an average thickness of ∼146 µm (Fig. S4(b)).

### 2.4 Material characterization

Morphological and electrochemical characterization of the R-TiO_2_ NTs/Ni(OH)_2_ NSs can be found in our previous work.^17^ For the characterization of the FLG and GNP nanostructures, transmission electron microscopy (TEM), scanning electron microscopy (SEM), and X-ray diffraction (XRD) were performed. Characterization of different ratios of Ti foil/FLG-GNP electrodes was analyzed by SEM (Zeiss EVO 10) operated at 20 kV. FLG and GNP nanostructures were analyzed by transmission electron microscopy (TEM) (JEM-2010, JEOL Ltd), operated at 200 kV. Structural analysis was carried out by X-ray diffraction (XRD) using a Panalytical XRD-6100 system with Cu Kα radiation of 1.54 Å, 40 kV, 30 mA as an X-ray source. Measurements were collected between 20° to 80° for 2*θ* with a step size of 0.0263°. Raman spectra of the FLG and GNP were recorded upon 532 nm laser excitation at room temperature (Renishaw InViaQontor). For all measurements, a 1800 l mm^−1^ grating was used. All data acquisition was performed using the Renishaw WiRE v.5.6 software package. The optical microscope photograph of the side view of the ASC composed of R-TiO_2_ NTs/Ni(OH)_2_ NSs electrode, Ti foil/FLG-GNP electrode, and the PVA/KOH gel electrolyte was obtained by using a Nikon LV100ND optical microscope.

Electrochemical testing was carried out on a Metrohm Autolab PGSTAT204 workstation in both three-electrode and two-electrode configurations. For three-electrode characterization, 1 M KOH aqueous solution served as the electrolyte. The fabricated Ti foil/FLG-GNP electrodes were used as the working electrode, while a SCE and a Pt sheet acted as the reference and counter electrodes, respectively. CV, GCD, and EIS were employed to evaluate performance. CV measurements were recorded in the potential range of −0.72 V to −0.2 V at scan rates of 10, 25, 50, and 100 mV s^−1^. GCD measurements were conducted to determine the charge storage behavior and areal specific capacitance of the electrodes. Measurements were conducted in the potential window of −0.6 V to 0 V at the current densities from 5 mA cm^−2^ to 0.5 mA cm^−2^. EIS studies were performed in the frequency range of 100 kHz to 10 mHz at a potential of 10 mV.

For the two-electrode configuration, PVA/KOH gel acted as both electrolyte and separator. Before device assembly, the charge balance between positive and negative electrodes was established to ensure optimal performance, according to eqn (1):^26^

1 *Q*_+_ = *Q*_−_

where the capacity is associated to the areal capacitance of the electrodes (*C*_s_), potential window (Δ*V*), and the electrode area (*A*) as shown in eqn (2)

2 *Q* = *C*_s_*×* Δ*V* × *A*

By combining (1) and (2), the following conditions must be satisfied:3
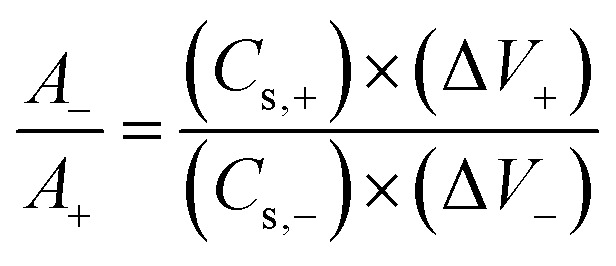


Since both electrodes had an exposed surface area of 1 cm^2^, the area ratio was approximately equal to unity.

In the two-electrode mode, SSC and ASC devices were characterized. Measurements for the SSC design was established by using R-TiO_2_ NTs/Ni(OH)_2_ NSs as both the positive and negative electrodes. For the characterization of the ASC design, the R-TiO_2_ NTs/Ni(OH)_2_ NSs electrode served as the positive electrode, while the negative electrode was the Ti foil/FLG-GNP. For all two-electrode measurements PVA/KOH gel was used as an electrolyte and separator. CV was carried out in the potential range of 0–1.6 V at scan rates ranging from 10 to 200 mV s^−1^. GCD curves were collected in the same potential window at current densities of 0.25 to 2.5 mA cm^−2^. EIS was again performed in the 100 kHz–10 mHz range with a 10 mV amplitude.

The specific areal capacitance (*C*_s_, mF cm^−2^) for the three-electrode configuration and for the two-electrode configuration was calculated from the GCD curves according to eqn (4) and (5) below:^27^4
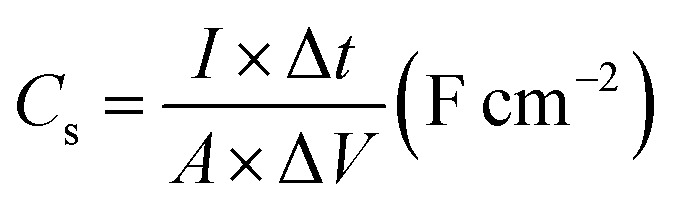
and5
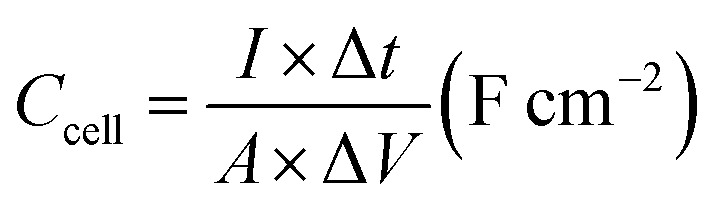
*I* (A) represents the applied discharge current, Δ*t* (s) is the discharging time, *A* (cm^2^) is the electrode's area, and Δ*V* (V) is the potential window.

For the supercapacitor device, energy density in areal *E*_D_ (µWh cm^−2^) and the power density *P*_D_ (mW cm^−2^) were evaluated using the equations below:^27^6
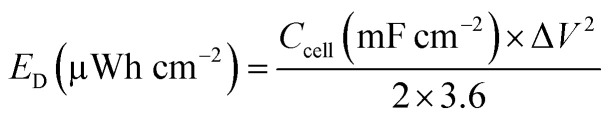
7
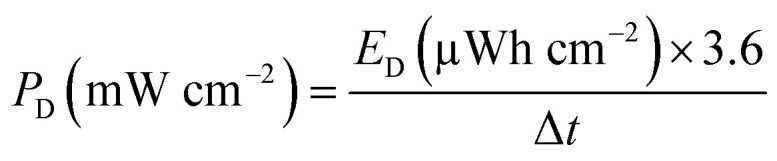


## 3 Results and discussion

**Fig. 1:**
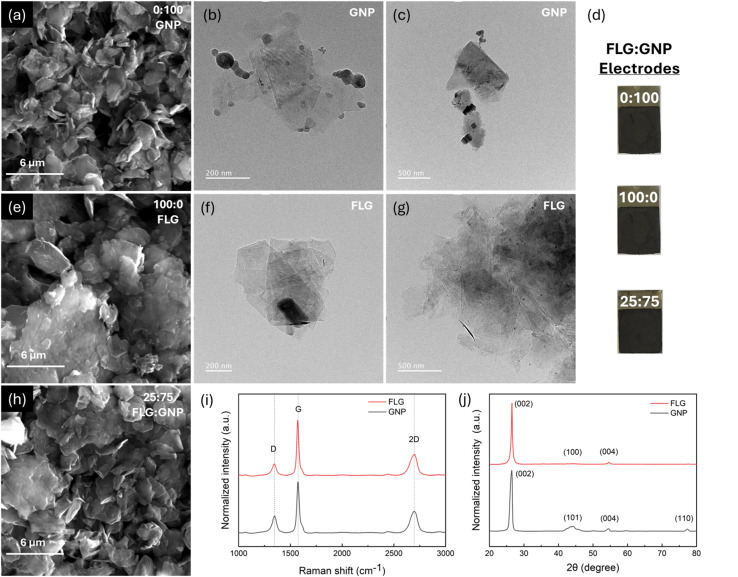
(a) SEM image of the only-GNP electrode with an FLG : GNP ratio of 0 : 100 (Ti foil/GNP). (b) and (c) TEM images of GNP under different magnifications. (d) Photographs of the Ti foil/GNP(0 : 100), Ti foil/FLG(100 : 0) and the Ti foil/FLG-GNP (25 : 75) electrodes. (e) SEM image of the only-FLG electrode with an FLG : GNP ratio of 100 : 0 (Ti foil/FLG). (f) and (g) TEM images of FLG under different magnifications. (h) SEM image of the FLG-GNP electrode with a FLG : GNP ratio of 25 : 75 (Ti foil/FLG-GNP). (i) Normalized and averaged Raman spectra and (j) normalized XRD pattern of FLG and GNP samples.

In the ASC configuration, the Ti foil/FLG-GNP electrode serves as the negative (anode) electrode. Its morphology was examined by SEM and TEM to evaluate the surface structure and nanosheet arrangement. Fig. 1(a) displays SEM images of the Ti foil/GNP electrode fabricated with a 0 : 100 FLG : GNP ratio. The surface shows platelet-like nanosheets with relatively large lateral dimensions forming a stacked network, which is typical of graphene nanoplatelet assemblies. TEM images of GNP (Fig. 1(b) and (c)) confirm the presence of thin, partially overlapped nanosheets with lateral sizes ranging from a few hundred nanometers to below one micrometer. Darker contrast regions correspond to thicker or agglomerated flakes, which is expected from van der Waals-driven restacking. Fig. 1(d) presents the photographic images of the fabricated electrode samples. All electrodes exhibit a similar appearance, indicating consistent preparation and uniform macroscopic characteristics across different samples. Fig. 1(e) displays the SEM image of the electrode containing only FLG (100 : 0 FLG : GNP). Compared with the GNP electrode, the FLG morphology reveals thinner, wrinkled, and less compact nanosheets, which provide a more open structure. TEM images of FLG shown in Fig. 1(f) and (g) further support this observation. The images illustrate multiple transparent, few-layer sheets with irregular edges and a high degree of folding. The contrast difference confirms the ultrathin nature of the flakes, which are stacked loosely compared to GNP. The wrinkled morphology is advantageous as it can prevent complete restacking of the nanosheets, thereby improving ion accessibility and enhancing electrochemical performance.

For the mixed composition, Fig. 1(h) presents the SEM image of the 25 : 75 FLG : GNP electrode. The image reveals a hybrid structure where thinner FLG sheets are distributed among the thicker GNP platelets. This configuration produces a more heterogeneous surface, combining the structural stability of GNP with the open architecture provided by FLG. Such hybrid morphology is anticipated to facilitate both electron transport through GNP networks and ion diffusion *via* the interlayer spacing of FLG. Additional SEM images of electrodes with other FLG : GNP ratios are provided in Fig. S1.

Raman scattering has been widely established as a fingerprint technique for determining the number of graphene layers.^28^Fig. 1(i) shows the normalized and averaged Raman spectra of FLG and GNP. The obtained spectra provide structural information about the materials, such as the presence of defects and the number of layers.^29^ For both materials, characteristic peaks of the graphitic materials, D (∼1350 cm^−1^), G (∼1570 cm^−1^), and 2D (∼2700 cm^−1^) are clearly observed. D peak represents the disordered mode of vibration, G peak corresponds to the ordered sp^2^ carbon–carbon bond mode of vibration in graphene, and 2D peak corresponds to a high-frequency phonon mode associated with the graphene sheets.^30^ The intensity ratio between the D and G bands, *I*(D)/*I*(G), is commonly used to evaluate defect density.^31^ The *I*(D)/*I*(G) values of 0.33 for FLG and 0.20 for GNP suggest a low to moderate number of defects (*I*(D)/*I*(G) < 0.5).^29,32^ The lower *I*(D)/*I*(G) ratio of GNP indicates fewer defects compared to FLG.^31,32^ On the other hand, the intensity ratio between the 2D and G bands, *I*(2D)/*I*(G), is associated with the number of layers.^31^ A high *I*(2D)/*I*(G) ratio is characteristic of monolayer or bilayer graphene, while values below 0.6 are generally associated with multilayer films (>4 layers), with values below 0.4 strongly indicating more than 5 layers.^32,33^ For both structures 2D peak had lower intensity than the G peak, indicating the materials consisted of more than one layer. The calculated *I*(2D)/*I*(G) ratio of 0.42 for the FLG sample suggests a structure of approximately 4–5 layers, whereas the value of 0.38 for GNP is consistent with a multilayered (5–10 layers) structure.^33^ Overall, the Raman spectra of FLG indicate the presence of a modest number of layers and a moderate level of defects, while the spectra of GNP correspond to a thicker multilayered structure with a lower defect density than FLG.

For direct comparison, XRD patterns of FLG and GNP were normalized with respect to the maximum intensity of the (002) peak, as shown in Fig. 1(j). The FLG sample exhibited diffraction peaks at approximately 26.5°, 44.4°, and 54.6°, corresponding to the (002), (100), and (004) planes of the graphene, respectively.^34^ In contrast, the GNP sample displayed characteristic diffraction peaks at approximately 26.3°, 44.3°, 54.5°, and 77.5°, attributed to the (002), (101), (004), and (110) planes.^35^ For FLG and GNP, a slight shift in the (002) peak is ascribed to an increase in the interlayer spacing relative to the natural graphite (interlayer spacing = 0.335 nm).^36^ According to Bragg's law, interlayer spacing was found to be 0.336 nm and 0.339 nm for FLG and GNP, respectively. For GNP, the (002) peak has a larger FWHM at lower diffraction angles, which is an indication of a larger crystallite size along the *c*-axis.^28^ Correlating well with the Raman studies, this indicates a wider arrangement of stacked layers in the GNP structure, as compared to FLG and regular graphite.^37^ Such expanded interlayer spacing of GNP is often found to be a desirable property, linked to improved electrical conductivity and facile intercalation of ions and molecules in energy storage applications.^28,38^

### 3.1 Electrochemical evaluation of Ti foil/FLG-GNP electrodes in a three-electrode (half-cell) configuration

The electrochemical behavior of all fabricated R-TiO_2_ TNs/Ni(OH)_2_ NSs electrodes, intended for the positive electrode in the ASC and SSC configurations, was systematically examined using CV, GCD, and EIS in a three-electrode (half-cell) setup with a standard counter and reference electrode. In our previous work, we demonstrated that the R-TiO_2_ TNs/Ni(OH)_2_ NSs hybrid electrode delivered an exceptionally high areal capacitance of 305.91 mF cm^−2^ at a current density of 0.75 mA cm^−2^, which significantly exceeded that of pristine TiO_2_ NTs (0.03 mF cm^−2^), R-TiO_2_ NTs (1.18 mF cm^−2^), and TiO_2_ NTs/Ni(OH)_2_ NSs (0.081 mF cm^−2^).^17^ This superior performance underscores the synergistic effect between oxygen vacancies in the R-TiO_2_ NTs substrate and the enhanced electrodeposition of Ni(OH)_2_ NSs on the more conductive framework. Owing to their high capacitance and excellent cycling stability, the R-TiO_2_ NTs/Ni(OH)_2_ NSs are highly promising as a positive electrode material.

To assemble the ASC, a negative electrode based on FLG-GNP nanostructures on Ti foil was fabricated. Although carbon nanostructures have been studied previously, to the best of our knowledge, this is the first report employing an FLG-GNP composite as the negative electrode in combination with an R-TiO_2_ TNs/Ni(OH)_2_ NSs positive electrode within this device configuration. Ti foil/FLG-GNP electrodes were prepared at various FLG : GNP weight ratios (0 : 100, 25 : 75, 50 : 50, 75 : 25, and 100 : 0) and evaluated under identical electrochemical conditions to reveal the individual and synergistic contributions of FLG and GNP to charge storage, conductivity, and rate capability. The 100 : 0 and 0 : 100 compositions served as baselines for the single components, while intermediate ratios (25 : 75, 50 : 50, 75 : 25) were employed to probe synergistic effects.

**Fig. 2:**
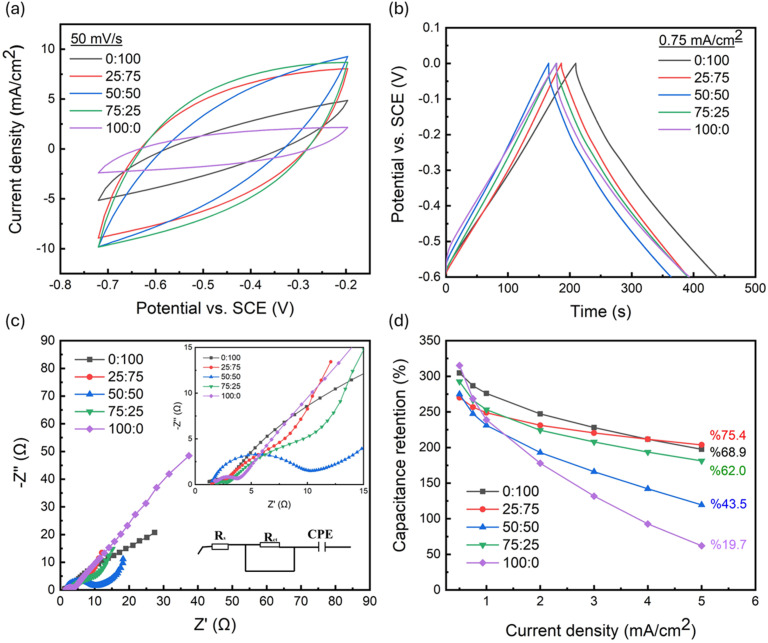
Comparison of the Ti foil/FLG-GNP electrodes prepared at different FLG to GNP (FLG : GNP) ratios (wt%). Comparison of the (a) CV curves at 50 mV s^−1^, (b) GCD curves at a current density of 0.75 mA cm^−2^, and (c) Nyquist plot in the frequency range of 100 kHz to 0.1 Hz with an amplitude of 5 mV. (d) Comparison of the capacitance retention of the Ti foil/FLG-GNP electrodes as a function of current density.

Fig. S2 reveals the scan rate study of each Ti foil/FLG-GNP electrode within −0.72 V to −0.2 V. All CV curves exhibit the typical rectangular shape characteristic of EDLCs, indicating that the charge is primarily electrostatic at the electrode/electrolyte interface. The CV area increased with the scan rate for all electrodes. At high scan rates, ion transport limitations caused a “leaf-like” distortion, whereas at lower scan rates (100 mV s^−1^ to 10 mV s^−1^) the curves approached an ideal rectangular profile. This behavior reflects the interplay between internal resistance, porosity, and ionic mobility.^39^ Notably, the electrode with a FLG : GNP ratio of 25 : 75 produced the largest CV area at 50 mV s^−1^ (Fig. 2(a)), implying superior charge storage capability.

Further, GCD tests performed in the −0.6 V to 0 V window at current densities of 5–0.5 mA cm^−2^ confirmed the EDLC nature of all Ti foil/FLG-GNP electrodes, with nearly triangular charge–discharge profiles (Fig. S3). Slight variations in discharge time at 0.75 mA cm^−2^ allowed calculation of areal capacitances: 286.62, 256.75, 247.5, 266.43, and 268.69 mF cm^−2^ for FLG : GNP ratios of 0 : 100, 25 : 75, 50 : 50, 75 : 25, and 100 : 0, respectively. As can be seen in Fig. 2(b), the highest capacitance was obtained for the pure GNP electrode (0 : 100), emphasizing the critical role of GNP in charge storage as well as interfacial kinetics. In contrast, the 50 : 50 electrode exhibited the lowest capacitance, confirming that equal FLG and GNP fractions provide a suboptimal platform for both charge transfer and storage.

EIS measurements further revealed compositional effects on interfacial charge transfer (Fig. 2(c)). The Ti-foil/FLG-GNP electrode at 50 : 50 showed the largest semicircle in the Nyquist plot, indicating slow interfacial kinetics. Apart from this composition, increasing the GNP proportion progressively reduced the semicircle diameter, highlighting the positive role of GNP in accelerating charge transfer. For 25 : 75 and 75 : 25 electrodes, two distinct semicircle features emerged: one at high frequencies (attributed to charge-transfer resistance) and a second, depressed semicircle at medium frequencies (indicative of finite-layer diffusion). Such mixed-type diffusion is commonly associated with concentration gradients of electroactive species in porous materials.^40,41^ To gain deeper insight into these observations, the Nyquist plots were fitted using an equivalent circuit model comprising *R*_s_, *R*_ct_, CPE (inset Fig. 2(c)). Here, *R*_s_ represents the solution resistance arising from the electrolyte and electrode contact; *R*_ct_ denotes the charge-transfer resistance at the electrode–electrolyte interface; and CPE (constant phase element) accounts for non-ideal capacitive behavior caused by surface roughness and heterogeneity. The fitted curves closely matched the experimental data, confirming the suitability of this model. An *R*_ct_ value of 3.91 Ω indicates a gradual decrease in charge-transfer resistance with increasing GNP content, reflecting enhanced interfacial conductivity and more efficient charge-transfer kinetics.

Since rate capability and long-term stability are vital for practical devices, capacitance retention at various current densities was monitored (Fig. 2(d)). Increasing the FLG fraction from 0% to 25% improved retention from 68.9% to 75.4%, but further increasing to 50% sharply reduced retention to 43.5%. A partial recovery (62.0%) occurred at 75 : 25. Notably, the pure FLG electrode (100 : 0) showed a drastic retention drop to 19.7%, again highlighting the pivotal role of GNP not only in charge storage but also in maintaining stability under high-rate conditions.^25^ Collectively, these results identify the 25 : 75 electrode as the optimal composition, combining high capacitance retention (75.4%), moderate areal capacitance (256.75 mF cm^−2^), and favorable charge-transfer resistance, making it a robust negative electrode for high-rate EDLC applications (Table S1).

Charge balance between the positive and negative electrodes is essential for efficient ASC operation. The R-TiO_2_ NTs/Ni(OH)_2_ NSs positive electrode exhibited 305.91 mF cm^−2^ at 0.75 mA cm^−2^ in 0–0.5 V,^17^ while the Ti-foil/FLG-GNP electrode with a FLG : GNP ratio of 25 : 75 delivered 256.75 mF cm^−2^ at 0.75 mA cm^−2^ in −0.6 to 0 V. Among all ratios tested, the 25 : 75 electrode yielded the closest *A*_−_/*A*_+_ ratio to unity (1.0071) per eqn (3), thus enabling ideal charge balance. In light of these findings, the FLG : GNP 25 : 75 composition was selected as the negative electrode for ASC fabrication due to its superior stability, balanced charge storage, and optimized interfacial kinetics.

### 3.2 Supercapacitor evaluation (full-cell characterization)

**Fig. 3:**
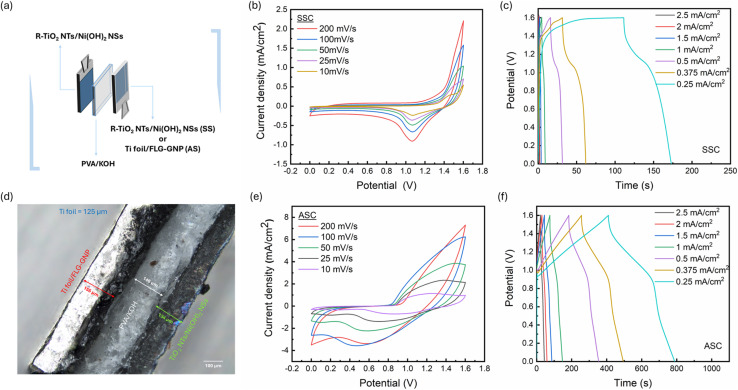
(a) Schematic representation of the SSC and ASC device fabrication based on two R-TiO_2_ NTs/Ni(OH)_2_ NSs electrodes as anode and cathode for the SSC fabrication, and R-TiO_2_ NTs/Ni(OH)_2_ NSs electrode as the cathode and Ti foil/FLG-GNP as the anode electrode for the ASC fabrication. Electrodes are sandwiched with a PVA/KOH gel electrolyte. (b) CV curves of SSC at different scan rates from 10 to 200 mV s^−1^ and (c) GCD curves at different current density values from 0.25 to 2.5 mA cm^−2^ in the potential window of 0 to 1.6 V. (d) Optical microscopy image of the ASC device showing the dimensions of the device components. (e) CV curves of the ASC device at different scan rates from 200 to 10 mV s^−1^ and (f) GCD curves of the ASC device from 0.25 to 2.5 mA cm^−2^ current density in the potential window of 0 to 1.6 V.

A schematic of the fabricated supercapacitor device (full cell) is shown in Fig. 3(a). For both SSC and ASC configurations, two electrodes were separated by a PVA/KOH gel electrolyte. The gel electrolyte was chosen instead of aqueous KOH to overcome the narrow electrochemical stability window of water, which typically leads to electrolyte degradation, gas evolution, and poor cycle life due to water electrolysis.^42^ For the SSC, two identical R-TiO_2_ NTs/Ni(OH)_2_ NSs electrodes served as positive and negative electrodes. For the ASC, the R-TiO_2_ NTs/Ni(OH)_2_ NSs electrode acted as the positive electrode, whereas a Ti foil/FLG-GNP electrode with an optimized FLG : GNP ratio of 25 : 75 functioned as the negative electrode. All devices were evaluated under a two-electrode configuration to determine their full-cell electrochemical characteristics.

Fig. 3(b) and (c) represent the CV and GCD measurements of the SSC, respectively. CV measurements were performed in the 0–1.6 V window at scan rates ranging from 10 mV s^−1^ to 200 mV s^−1^. As illustrated in Fig. 3(b), the CV profiles retained their shape with increasing scan rate, and the peak intensities in both anodic and cathodic directions increased proportionally, indicating stable redox behavior. The corresponding GCD curves in Fig. 3(c) display non-linear charge–discharge profiles consistent with the redox peaks observed in the CVs. At lower current densities, the charge–discharge times increased, reflecting higher specific capacitance due to more complete ion insertion/extraction. An optical micrograph of the assembled ASC, along with its dimensional layout, is presented in Fig. 3(d). Fig. S4(a) compares the CV curves of the individual positive (R-TiO_2_ NTs/Ni(OH)_2_ NSs) and negative electrode (Ti foil/FLG-GNP) at a scan rate of 50 mV s^−1^, confirming their complementary electrochemical windows. Fig. 3(e) and (f) display the CV and GCD profiles of the ASC at various scan rates and current densities, respectively. In contrast to the SSC, the ASC exhibits broader redox peaks, a larger enclosed CV area, and higher current response (Fig. 3(e)), demonstrating the enhanced charge storage capability imparted by the FLG-GNP negative electrode. This clear difference between SSC and ASC behavior highlights the beneficial role of FLG and GNP in accelerating charge transfer and enabling hybrid energy-storage mechanisms.

Specifically, the CV profiles of the ASC reveal the coexistence of faradaic (pseudocapacitive) and non-faradaic (EDLC) processes in a single device. The Ni(OH)_2_ NSs on R-TiO_2_ NTs provide dominant pseudocapacitance, whereas FLG and GNP contribute rapid, reversible double-layer charging. Consequently, the ASC GCD curves in Fig. 3(f) deviate from a purely triangular EDLC profile and display discernible plateau regions, which correspond to faradaic reactions and longer charge–discharge periods. Rate-capability tests were performed between 0.25 and 2.5 mA cm^−2^. At higher current densities, faster discharge profiles were observed because ions had insufficient time to diffuse through the electrode network. Conversely, at lower current densities, the plateau region extended, allowing deeper ion penetration and yielding higher capacities.^43,44^ Taken together, these results confirm that integrating R-TiO_2_ NTs/Ni(OH)_2_ NSs with the optimized FLG-GNP electrode in an ASC configuration produces a synergistic effect: pseudocapacitance from Ni(OH)_2_ NSs combined with rapid EDLC behavior from FLG and GNP. This hybrid mechanism delivers superior charge storage, improved rate performance, and enhanced cycling stability compared with the symmetric device.^45^

**Fig. 4:**
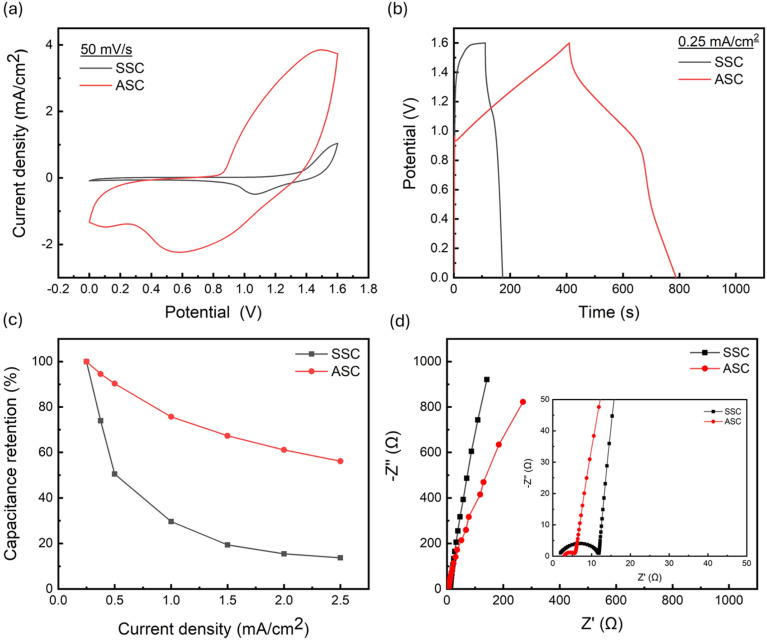
Comparison of the (a) CV curves at 50 mV s^−1^ and (b) GCD curves at 0.25 mA cm^−2^ in the potential window from 0 to 1.6 V for the SSC (black) and ASC (red) devices. (c) Capacitance retention of the SSC (black) and ASC (red) devices as a function of current density. (d) Comparison of the Nyquist plot of the SSC and ASC devices in the frequency range of 100 kHz to 0.1 Hz with an amplitude of 5 mV (inset showing the enlarged portion of the high frequency region).

A direct comparison of the SSC and ASC devices is presented in Fig. 4. The CV curves at 50 mV s^−1^ (Fig. 4(a)) clearly show that the ASC has a larger enclosed CV area and higher current density than the SSC. Consistently, the GCD profiles at 0.25 mA cm^−2^ (Fig. 4(b)) reveal that the ASC exhibits a markedly longer discharge time than the SSC. Together, these results indicate a substantially higher areal specific capacitance for the ASC (118.26 mF cm^−2^) compared with the SSC (19.38 mF cm^−2^) at 0.25 mA cm^−2^. This improvement demonstrates how the intrinsic voltage and capacitance limitations of SSCs, arising from the use of identical electrode materials, are effectively overcome by employing an ASC configuration. In the present design, the positive electrode (R-TiO_2_ NTs/Ni(OH)_2_ NSs) provides pseudocapacitance behavior, while the negative electrode (Ti-foil/FLG-GNP) contributes. The combination of two different active materials operating in complementary potential windows significantly enhances both areal capacitance and energy density compared to the SSC.^46^

For practical applications, energy and power densities are key parameters in evaluating supercapacitor performance. Using eqn (6) and (7), these values were determined for both SSC and ASC devices. At a current density of 0.25 mA cm^−2^, the SSC exhibited an energy density of 6.89 µWh cm^−2^, while the ASC reached 42.05 µWh cm^−2^, corresponding to an approximate six-fold improvement. As revealed in Table S2, comparison with previously reported SSCs based on anatase TiO_2_ NTs shows that the R-TiO_2_ NTs/Ni(OH)_2_ electrode provides a 13.6-fold higher specific capacitance, a 38.3-fold increase in energy density, and a three-fold enhancement in power density. Among recently developed ASC configurations, the R-TiO_2_ NTs/Ni(OH)_2_ ‖ Ti-foil/FLG-GNP system demonstrates particularly promising performance, delivering both higher energy density and areal capacitance than other reported designs, highlighting its potential for advanced energy storage applications.

The superior performance of the ASC originates from the unique microstructure of the FLG-GNP composite, which enhances the accessible surface area and promotes rapid ion transport. This synergistic effect is reflected in the rate-capability data presented in Fig. 4(c). As the current density increased, the SSC experienced a sharp decline in capacitance, retaining only 20% of its initial value at 1.5 mA cm^−2^ and 13% at 2.5 mA cm^−2^. In contrast, the ASC retained 70% of its initial capacitance at 1.5 mA cm^−2^ and nearly 60% at 2.5 mA cm^−2^, despite the current density being ten times higher than that used for the initial measurement (0.25 mA cm^−2^). This comparison clearly demonstrates that the ASC possesses a significantly higher rate capability than the SSC.

These electrochemical trends are further supported by impedance analysis (Fig. 4(d)). Both Nyquist plots show a semicircle in the high-frequency region (indicative of charge-transfer resistance) followed by an almost vertical line at low frequencies (indicative of capacitive behavior). The semicircle diameter is substantially larger for the SSC, reflecting higher interfacial resistance that hinders ion migration and lowers capacitance.^47^ By contrast, the ASC displays a smaller semicircle and a more pronounced vertical line, which correspond to lower charge-transfer resistance and enhanced double-layer capacitance at the electrode/electrolyte interface.^48^

These findings collectively highlight the advantages of the ASC design, where the combination of pseudocapacitance from Ni(OH)_2_ NSs and EDLC behavior from FLG-GNP contributes to enhanced electrochemical performance, including higher energy and power densities, improved rate capability, and greater stability compared to the SSC.

**Fig. 5:**
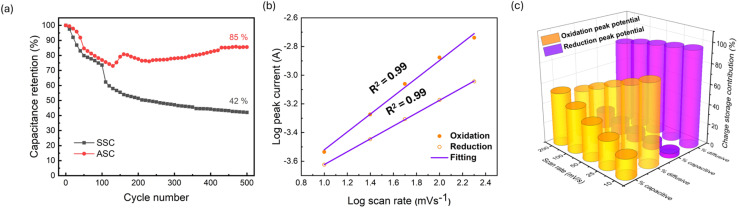
(a) Comparison of the capacitance retention of the SSC and ASC devices for 500 cycles at 100 mV s^−1^ scan rate. (b) Log–log plots of peak current *versus* scan rate for oxidation and reduction processes of the ASC device, showing slopes close to unity (*R*^2^ = 0.99), indicative of a surface-controlled capacitive mechanism. (c) Capacitive and diffusion-controlled contributions of oxidation and reduction processes at various scan rates, showing increasing capacitive dominance with higher scan rates.

The long-term stability of the supercapacitor devices was assessed through capacitance retention measurements at a scan rate of 100 mV s^−1^ (Fig. 5(a)). During the initial 150 cycles, both devices exhibited a decrease in capacitance; however, only the ASC showed subsequent recovery. For the SSC, the decline continued steadily, and by the 500th cycle, the areal capacitance had dropped by 58%, retaining just 42% of its initial value. In contrast, the ASC demonstrated a notable recovery after the initial decrease, which can be attributed to the activation of the electrode surfaces and the optimization of charge-transfer dynamics. Following this activation, the capacitance gradually increased and stabilized, ultimately maintaining 85% of its initial value over 500 cycles. This distinct difference between SSC and ASC performance highlights the enhanced stability and superior electrochemical behavior of the ASC, demonstrating the significant advantage of incorporating FLG-GNP as the anode material in asymmetric supercapacitor designs. The superior electrochemical performance of the ASC device can be further understood by examining the underlying charge storage mechanism. As shown in Fig. 5(b), the log–log relationship between peak current and scan rate exhibits slopes close to unity for both the anodic and cathodic processes (*R*^2^ ≈ 0.99). This indicates that the charge storage is predominantly governed by surface-controlled processes rather than diffusion-limited intercalation. Such behavior highlights the rapid and reversible faradaic reactions of the Ni(OH)_2_ nanosheets, which effectively contribute to pseudocapacitive charge storage. To further quantify the relative contributions of capacitive and diffusion-controlled processes, the current response was deconvoluted according to *i*(*V*) = *k*_1_*ν* + *k*_2_*ν*^1/2^, and the results are presented in Fig. 5(c). The 3D bar plot clearly demonstrates that, across increasing scan rates, capacitive contributions become increasingly dominant compared to diffusion-controlled contributions. At lower scan rates, ion diffusion into the electrode bulk plays a more notable role; however, as the scan rate increases, charge storage is almost entirely surface-driven. This mechanistic insight confirms that the ASC benefits from the synergistic contributions of Ni(OH)_2_ pseudocapacitance and FLG-GNP double-layer capacitance, thereby ensuring rapid charge–discharge kinetics, enhanced rate performance, and long-term cycling stability.

## 4 Conclusion

In this study, thin-film SSC and ASC supercapacitors based on R-TiO_2_ NT electrodes were successfully developed through a simple yet controlled fabrication process. The SSC was assembled with identical R-TiO_2_ NTs/Ni(OH)_2_ nanosheet electrodes, while the ASC design employed R-TiO_2_ NTs/Ni(OH)_2_ NSs as the cathode and Ti foil/FLG-GNP as the anode, integrated with a PVA/KOH gel electrolyte. Prior to ASC assembly, systematic evaluation of FLG-GNP composites identified the crucial role of GNP in improving electrode durability under high current loads, with the 25 : 75 (wt%) FLG : GNP ratio exhibiting the best performance, retaining 75.4% of its capacitance. Electrochemical testing showed that the ASC achieved an areal capacitance of 118.26 mF cm^−2^, approximately six times higher than the SSC (19.38 mF cm^−2^), along with nearly a seven-fold improvement in energy density (42.05 *vs.* 6.89 µWh cm^−2^). This outstanding performance is attributed to the synergistic contributions of EDLC from the FLG-GNP composite and pseudocapacitance from Ni(OH)_2_ NSs, which enable rapid charge storage, enhanced stability, and superior rate capability. Importantly, the superior performance of the ASC can be ascribed to a synergistic charge storage mechanism, where surface-controlled pseudocapacitive reactions of Ni(OH)_2_ nanosheets complement the double-layer capacitance of the FLG-GNP electrode, ensuring rapid charge–discharge kinetics, high rate capability, and excellent cycling stability. These results establish R-TiO_2_ NTs as a highly promising substrate for supercapacitor electrode design. More importantly, the demonstrated ASC configuration provides a valuable framework for combining EDLC and pseudocapacitive materials, opening new opportunities for TiO_2_ NT-based energy storage devices with high capacitance, energy density, and long-term cycling stability.

## Conflicts of interest

The authors declare no competing financial interests.

## Supplementary Material

RA-016-D5RA07750H-s001

## Data Availability

The data supporting this article have been included as part of the supplementary information (SI). Supplementary information: Tables S1 and S2, SEM images, CV curves, GCD curves and further experimental details. See DOI: https://doi.org/10.1039/d5ra07750h.
