# Supplementary material for: Enhanced energy storage in supercapacitors using R-TiO2 nanotube and graphene-based electrodes
Source: RSC Adv. 2026 Feb 6;16(9):7911–21. doi: 10.1039/d5ra07750h (PMC12879991; doi:10.1039/d5ra07750h)
Supplement: RA-016-D5RA07750H-s001 [file RA-016-D5RA07750H-s001.pdf]

Supplementary Information (SI) for RSC Advances.
This journal is © The Royal Society of Chemistry 2026

Supplementary Material

Enhanced Energy Storage in Supercapacitors Using R-TiO_2 Nanotube and Graphene-Based Electrodes

Sensu Tunca[a,b], Iqra Rabani[a,b], Karolien De Wael[a,b,*]

[a] Antwerp Engineering, Photoelectrochemistry & Sensing (A-PECS), University of Antwerp, Groenenborgerlaan 171, 2020 Antwerp, Belgium

[b] NANOlight Center of Excellence, University of Antwerp, Groenenborgerlaan 171, 2020 Antwerp, Belgium

* Corresponding author.

E-mail: karolien.dewael@uantwerpen.be

1. Experimental details

1.1 Chemicals

Temper-annealed Ti foil (99.6 +% purity, thickness 0.125 mm) was purchased from ADVENT Research Materials Ltd. (Oxford, UK). Ethylene glycol ($C_2H_6O_2$, analytical reagent) and N-methyl-2-pyrrolidinone (analytical reagent) were obtained from Sigma-Aldrich (USA). Potassium hydroxide (KOH, AR) was supplied by both Sigma-Aldrich (USA) and Chem-Lab NV (Zedelgem, Belgium). Ammonium fluoride (NH_4F, AR) was obtained from Merck (Darmstadt, Germany), while sodium sulfate (Na_2SO_4, AR) was obtained from Chem-Lab NV (Zedelgem, Belgium). Nickel(II) acetate tetrahydrate ($Ni(CH_3COO)_2 \cdot 4H_2O$, AR) was purchased from Thermo Fisher Scientific (Kandel, Germany). Active carbon was obtained from Merck (Darmstadt, Germany), poly(vinylidene fluoride) (PVDF) from Thermo Scientific (Kandel, Germany), and poly(vinyl alcohol) (PVA, $[-CH_2CH(OH)-]_n$, 98% hydrolyzed) from Aldrich-Chemie (Steinheim, Germany). FLG and GNP were purchased from TCI Deutschland GmbH (Germany) and used without further purification.

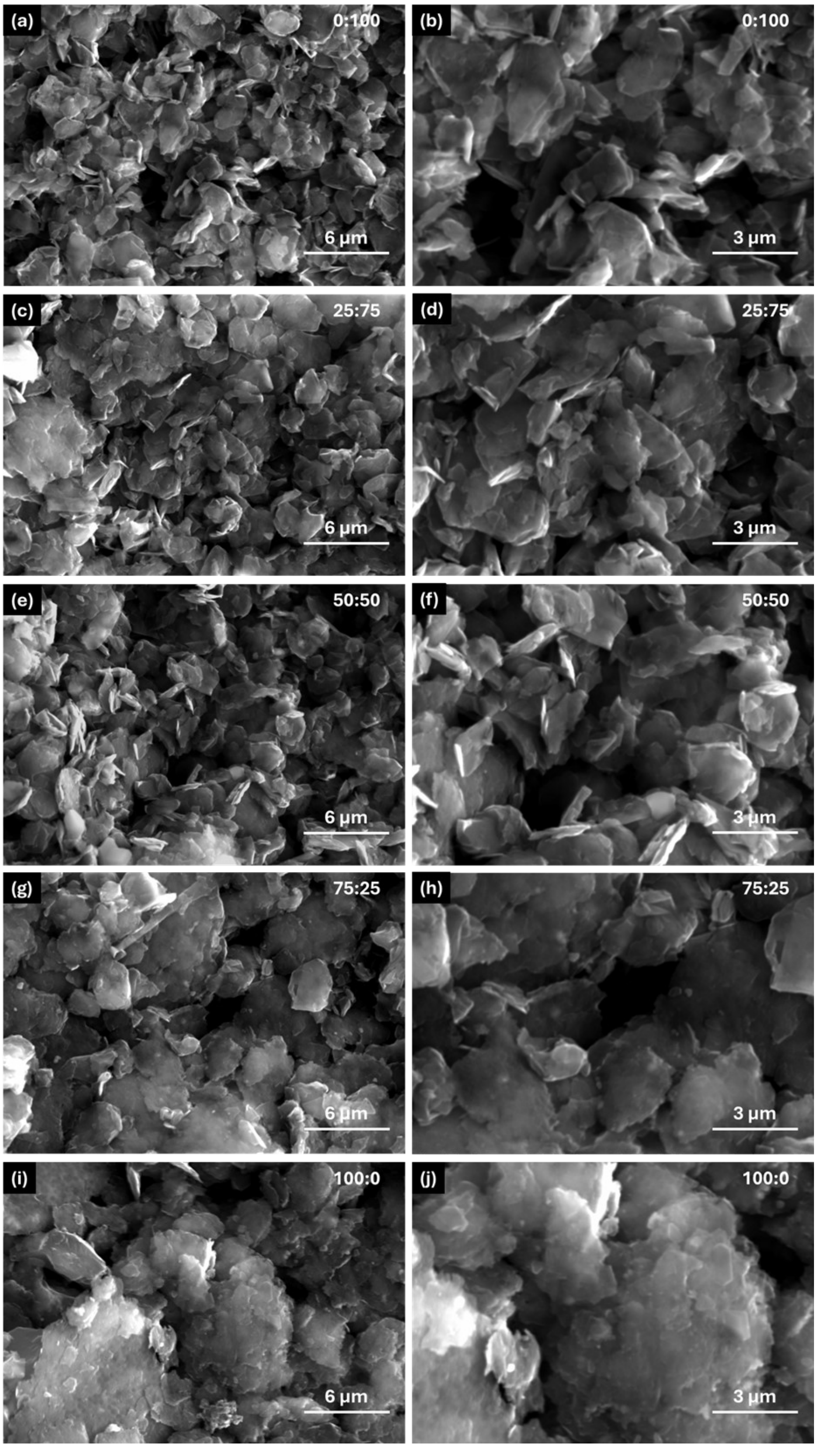

Figure S1. SEM images of the different ratio (in wt%) Ti foil/FLG:GNP electrodes (a)-(b) 0:100, (c)-(d) 25:75, (e)-(f) 50:50, (g)-(h) 75:25, (i)-(j) 100:0. SEM images in (a), (c), (e), (g), (j) were collected at a magnification of 10 kX, and (b), (d), (f), (h), (j) at a magnification of 20kX.

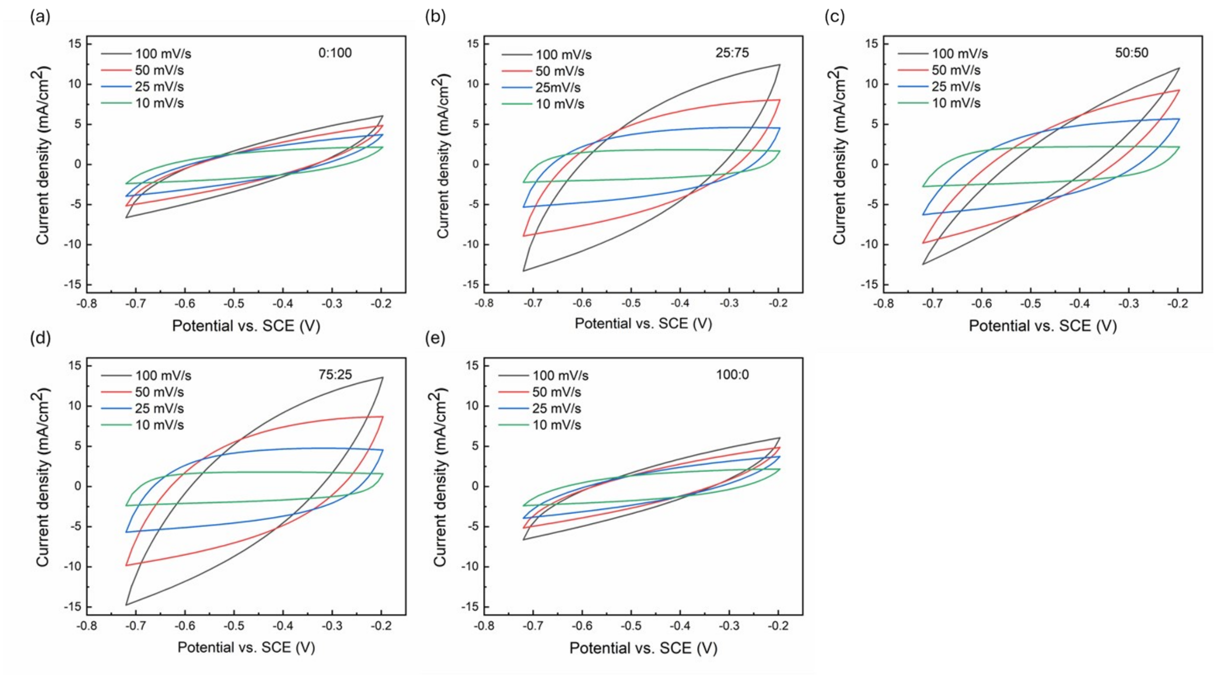

Figure S2. CV curves of the Ti foil/FLG-GNP electrodes having different FLG:GNP ratio (a) 0:100, (b) 25:75, (c) 50:50, (d) 75:25, (e) 100:0 in the potential window of -0.72 V to -0.2 V (vs. SCE) at different scan rates of 100 to 10 mV/s in 1M KOH electrolyte.

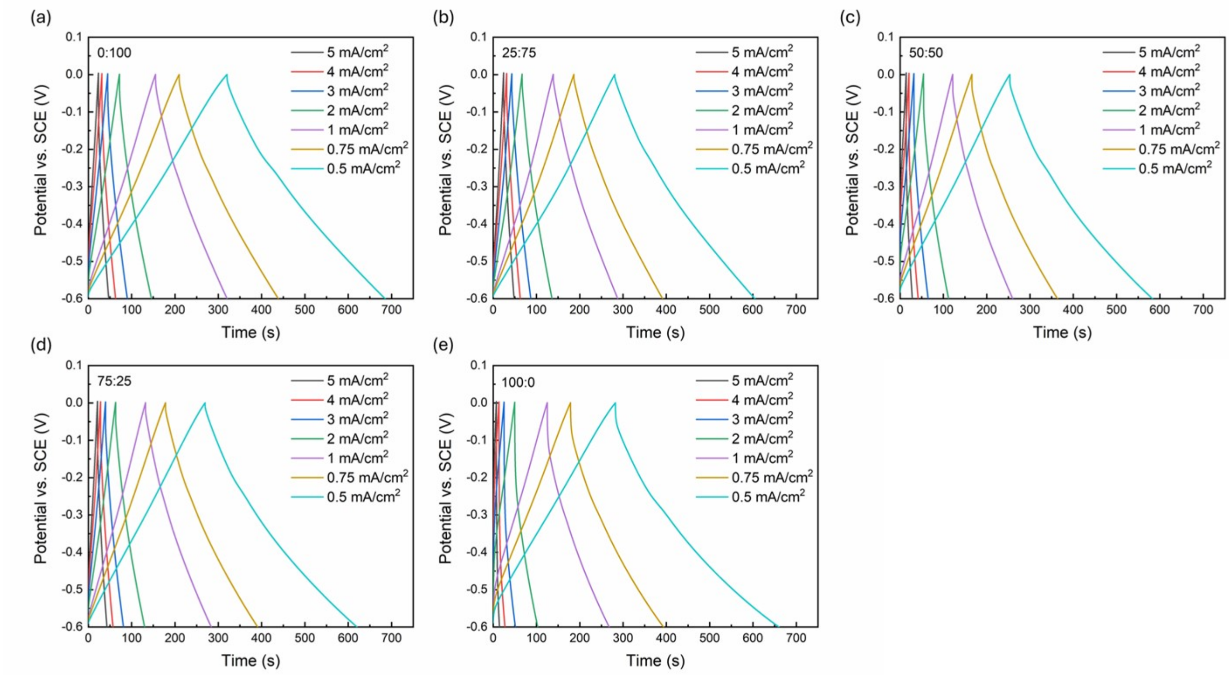

Figure S3. GCD curves of the Ti foil/FLG-GNP electrodes having different FLG:GNP ratio (a) 0:100, (b) 25:75, (c) 50:50, (d) 75:25, (e) 100:0 in the potential window of -0.6 V to 0 V (vs. SCE) at different current densities ranging from 5 to 0.5 mA/cm^2 in 1M KOH electrolyte.

Table S1. Summary of the performance of the Ti foil/FLG-GNP electrodes having different FLG to GNP (FLG:GNP) ratios.

FLG to GNP ratio (wt%) (FLG:GNP)	Areal capacitance (mF/cm^2) at 0.75 mA/cm^2	Capacitance retention (%) from 5 to 0.5 mA/cm^2
0:100	286.6	68.9
25:75	256.75	75.4
50:50	247.5	43.5
75:25	266.4	62.0
100:0	268.7	19.7

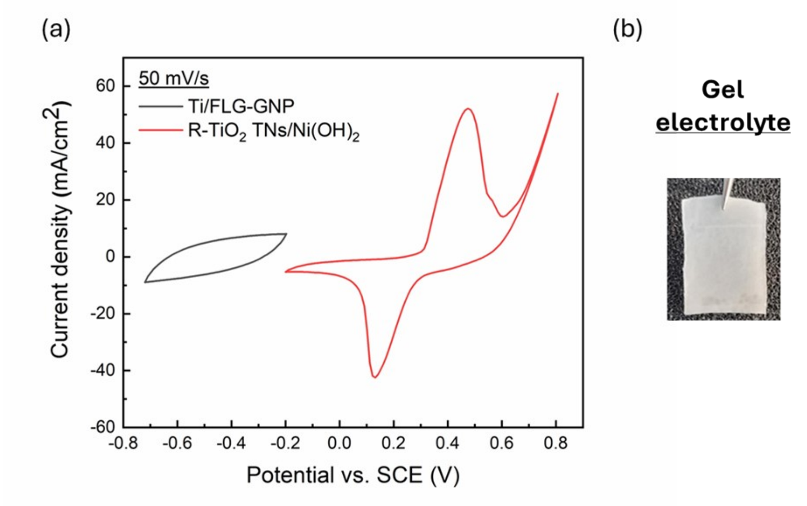

Figure S4. (a) CV curves of positive (R-TiO_2 NTs/ $Ni(OH)_2$ NSs) and negative electrode (Ti foil/FLG-GNP) at a scan rate of 50 mV/s in 1M KOH electrolyte.(b) Photographs of the gel electrolyte.

Table S2. Performance of the previously reported thin film supercapacitors.

Electrode material (SC type)	Areal capacitance (Csp, mF/cm^2)	Electrolyte	Potential window (V)	Energy density (Ed, $\mu Wh/cm^2$)	Power density (Pd, mW/cm^2)	Ref.
CNT/Ppy (SSC))	2185.1	PVA/H_2SO_4 gel	0 to 0.8 V	194.23	0.4	[1]
GWF/PANI (SSC)	23	PVA/H_3PO_4 gel	-0.5 to 0.5V	0.8	0.3	[2]
Anatase TiO_2 NTs (SSC)	1.42	Dulbecco's Phosphate Buffered Saline	-0.5 to 0.5V	0.18	0.136	[3]
$NiCo_2O_4$ on thin film Carbon layer (SSC)	42.6	PVA-KOH gel	0 to 1 V	-	-	[4]
V_2O_5/ZnO thin film (SSC)	83.59	LiCl/PVA gel	0 to 1 V	0.46	0.062	[5]
$NiCo_2O_4$ NW	161	PVA-KOH	0 to 1 V	-	-	[6]

array on Ni foam (SSC)		gel				
Fe-Doped MoS_2 nanosheets | AC (ASC)	430	3 M KOH	0 to 1.8 V	133	7.5	[7]
Hy-NiCoS/AC (ASC)	85	PVA-KOH gel	0 to 1 V	17	3.2	[8]
Lead-free perovskite-based thin film (ASC)	64	PVA/LiTFSi gel	0 to 1.6 V	6.6	~0.1	[9]
C- Fe_2O_3 NPs coated Ta_2O_5 NTs (SSC)	18.2	1 M TEA in acetonitrile	0 to 2.5 V	-	-	[10]
MXene/Fe_3O_4/MXene film (SSC)	46.4	1 M Li_2SO_4	0 to 0.7 V	0.970	0.176	[11]
V_2O_5– TiN (200−40)/Ag (SSC)	98.66	6 M KCl		30.83	3.0	[12]
V_2O_5 (SSC)	9.7	PVA/KOH gel	0 to 1 V	0.68	0.095	[13]
Ti_3C_2Tx MXene/SWCNT (ASC)	1.6	PVA/H_2SO_4	0 to 1 V	0.05	0.0024	[14]
R-TiO_2 NTs/$Ni(OH)_2$ | Ti foil/FLG-GNP(ASC)	118.26	PVA-KOH gel	0 to 1.6 V	42.05	0.4	This work
R-TiO_2 NTs/$Ni(OH)_2$ (SSC)	19.38	PVA-KOH gel	0 to 1.6 V	6.89	0.4	This work

References

1 L. Tong, M. Gao, C. Jiang and K. Cai, *J. Mater. Chem. A*, 2019, **7**, 10751–10760.

2 X. Zang, X. Li, M. Zhu, X. Li, Z. Zhen, Y. He, K. Wang, J. Wei, F. Kang and H. Zhu, *Nanoscale*, 2015, **7**, 7318–7322.

3 A. Lamberti and C. F. Pirri, *J. Energy Storage*, 2016, **8**, 193–197.

4 Y. Ai, J. Ma and Z. M. Wang, *IOP Conf. Ser. Earth Environ. Sci.*, 2018, **170**, 032095.

5 M. Karuppaiah, J. K. Lee and G. Ravi, *ACS Appl. Electron. Mater.*, 2024, **6**, 1504–1513.

6 Q. Wang, X. Wang, B. Liu, G. Yu, X. Hou, D. Chen and G. Shen, *J. Mater. Chem. A*, 2013, **1**, 2468.

7 P. J. Morankar, T. V. M. Sreekanth, R. U. Amate, M. A. Yewale, A. M. Teli, S. A. Beknalkar and C.-W. Jeon, *Coatings*, 2024, **14**, 1328.

8 S.-F. Tseng, J.-Y. Lin and J.-Y. Lin, *Energy*, 2024, **291**, 130365.

9 A. Yadav, A. Saini, P. Kumar and M. Bag, *J. Mater. Chem. C*, 2024, **12**, 197–206.

10 Y. Yang, Z. Peng, G. Wang, G. Ruan, X. Fan, L. Li, H. Fei, R. H. Hauge and J. M. Tour, *ACS Nano*, 2014, **8**, 7279–7287.

11 H. Li, Y. Liu, S. Lin, H. Li, Z. Wu, L. Zhu, C. Li, X. Wang, X. Zhu and Y. Sun, *J. Power Sources*, 2021, **497**, 229882.

12 V. Raman, N. Clament Sagaya Selvam, S. B. Mitta and H.-K. Kim, *J. Alloys Compd.*, 2024, **971**, 172450.

13 R. Velmurugan, J. Premkumar, R. Pitchai, M. Ulaganathan and B. Subramanian, *ACS Sustain. Chem. Eng.*, 2019, **7**, 13115–13126.

14 C. (John) Zhang, B. Anasori, A. Seral-Ascaso, S. Park, N. McEvoy, A. Shmeliov, G. S. Duesberg, J. N. Coleman, Y. Gogotsi and V. Nicolosi, *Adv. Mater.*, DOI:10.1002/adma.201702678.
